# Mast Cell Chymase and Kidney Disease

**DOI:** 10.3390/ijms22010302

**Published:** 2020-12-30

**Authors:** Shamila Vibhushan, Manuela Bratti, Juan Eduardo Montero-Hernández, Alaa El Ghoneimi, Marc Benhamou, Nicolas Charles, Eric Daugas, Ulrich Blank

**Affiliations:** 1 Centre de Recherche sur l’inflammation, CNRS ERL8252, Faculté de Médecine site Bichat, Université de Paris, Inserm UMR1149, 16 rue Henri Huchard, F-75018 Paris, France; shamila.vibhushan@inserm.fr (S.V.); manuela.bratti@inserm.fr (M.B.); juan-eduardo.montero-hernandez@inserm.fr (J.E.M.-H.); alaa.elghoneimi@rdb.aphp.fr (A.E.G.); marc.benhamou@inserm.fr (M.B.); nicolas.charles@inserm.fr (N.C.); eric.daugas@sls.ap-hop-paris.fr (E.D.); 2 Laboratoire d’Excellence Inflamex, Université de Paris, F-75018 Paris, France; 3 Department of Pediatric Surgery and Urology, Hôpital Universitaire Robert Debré, Assistance Publique—Hôpitaux de Paris (APHP), F-75019 Paris, France; 4 Service de Néphrologie, Groupe Hospitalier Universitaire Bichat-Claude Bernard, Assistance Publique—Hôpitaux de Paris (APHP), F-75019 Paris, France

**Keywords:** kidney disease, angiotensin II, inflammation, mast cell, mast cell chymase

## Abstract

A sizable part (~2%) of the human genome encodes for proteases. They are involved in many physiological processes, such as development, reproduction and inflammation, but also play a role in pathology. Mast cells (MC) contain a variety of MC specific proteases, the expression of which may differ between various MC subtypes. Amongst these proteases, chymase represents up to 25% of the total proteins in the MC and is released from cytoplasmic granules upon activation. Once secreted, it cleaves the targets in the local tissue environment, but may also act in lymph nodes infiltrated by MC, or systemically, when reaching the circulation during an inflammatory response. MC have been recognized as important components in the development of kidney disease. Based on this observation, MC chymase has gained interest following the discovery that it contributes to the angiotensin-converting enzyme’s independent generation of angiotensin II, an important inflammatory mediator in the development of kidney disease. Hence, progress regarding its role has been made based on studies using inhibitors but also on mice deficient in MC protease 4 (mMCP-4), the functional murine counterpart of human chymase. In this review, we discuss the role and actions of chymase in kidney disease. While initially believed to contribute to pathogenesis, the accumulated data favor a more subtle view, indicating that chymase may also have beneficial actions.

## 1. Introduction

Chymase is a highly abundant mast-cell (MC) specific serine protease that is synthesized as a pro-peptidase and activated by dipeptidyl peptidase I (also known as cathepsin C) before being stored in a releasable form bound to highly negatively charged proteoglycans in MC cytoplasmic granules [1]. While human MCs have only one chymase (encoded by *CMA1*), mice express several chymases, including murine MC protease 1 (mMCP-1), mMCP-2, mMCP-4, mMCP-5 and mMCP-9, in different MC types. Although not the enzyme with the highest sequence homology to human α-chymase, mMCP-4, a β-chymase, most likely reflects its functional counterpart exhibiting a similar tissue distribution in connective tissue-type MC and similar chymotryptic cleavage properties [2,3,4]. Human chymase, as well as mMCP-4 and -5, are negatively charged and stored in MC granules in a complex with granular proteoglycans. By contrast, storage of the less negatively charged mMCP-1 is proteoglycan-independent, with an intermediate phenotype being observed for mMCP-2 [5]. When MC degranulate, proteoglycan-bound chymase is dispersed into the interstitial spaces that remain associated to proteoglycans, which protects it from its natural inhibitors (such as α1-antitrypsin, α2-antichymotrypsin, α-macroglobulin). Proteoglycan binding also limits its diffusion, allowing the enzyme to remain locally active in the long-term after release [6]. However, contrary to the notion that chymase acts only locally [3,7], new studies suggest that chymase also has systemic functions under inflammatory conditions [8,9,10].

Human chymase cleaves peptides after aromatic amino acids (Phe, Tyr, Trp and sometimes Leu) in a variety of both endogenous and exogenous peptides and protein targets, albeit not all of them have been confirmed in vivo [4,11]. Some of these targets could also play a role in renal inflammatory diseases. In particular, human as well as some rodents’ chymases have been known for long to play a role in the generation of Angiotensin II (Ang II) from the Angiotensin I precursor [12,13,14] in tissues, including in the kidney. Thus, chymase may generate Ang II independently from the angiotensin-converting enzyme (ACE), one of the central enzymes of the classical renin-angiotensin system (RAS), which is an important regulator of intrarenal hemodynamics, glomerular filtration, as well as fluid and electrolyte homeostasis [15]. This generation of Ang II by chymase in the kidneys was proposed as an important compensatory mechanism to maintain a steady-state Ang II formation in ACE knockout mice [16]. Another protease released by MCs, MC carboxypeptidase A (CPA), may play an additional role in the generation of Ang II and some antagonistic peptides [17]. Evidence based on the use of chymase inhibitors, or on studies in MC-deficient mice, clearly confirm that blood pressure regulation can also involve an ACE-independent intrarenal system of Ang II generation [18,19,20]. In addition to its Ang II generating activity, chymase was also shown to activate a number of pro-peptides involved in the inflammatory and tissue remodeling response, such as the latent transforming growth factor-β (TGF-β) [21,22], IL-33 [23], pro-matrix metalloproteinases (MMP) [24,25] and big endothelin 1 (the precursor of endothelin 1) [26,27]. There is also substantial evidence for its implication in the coagulation system, as it is capable to degrade thrombin and plasmin [28,29,30]. It also degrades proteins of the extracellular matrix, such as fibronectin [30,31], and a variety of other inflammatory proteins, including a select set of cytokines and adhesion receptors [10,11,32].

**Figure 1:**
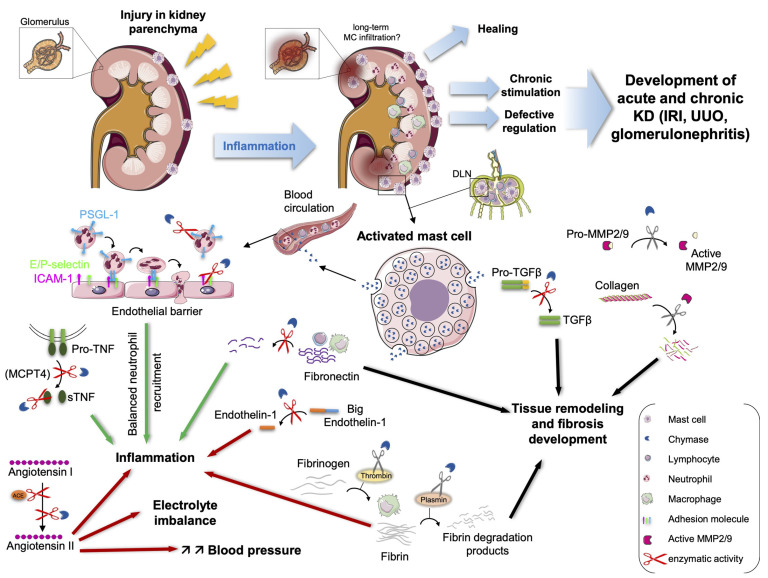
Proposed functions of chymase in kidney disease according to the explications provided in the text. The red scissors indicate enzymatic activity in KD, as demonstrated in scientific publications, while the grey scissors indicate possible activities in KD. The green arrows indicate positive protective functions of chymase. The red arrows indicate disease-aggravating functions of chymase.

Kidney disease (KD) is a growing health problem worldwide with little treatment options so far, except for replacement therapy [33]. Although some forms of the disease are of genetic origin (i.e., polycystic KD), KD is generally initiated by an inflammatory process that starts with an injury that can be of toxic, metabolic (diabetes), hemodynamic (hyper-, hypo-tension), post-ischemic, infectious, autoimmune… origin, affecting the kidney parenchyma, and particularly the glomeruli, renal vessels or tubulo-interstitial compartment (Figure 1). The initial injury launches an inflammatory cascade which, in case of chronic stimulation or defective regulation, enters into a progressive phase, with the development of chronic KD (CKD) engendering the destruction of individual nephrons and blood vessels and finally end-stage renal failure (Figure 1). The RAS system leading to the generation of Ang II has been recognized as a major factor of disease progression and renal fibrosis development. In medical care, the relentless decline in renal function can be slowed down by therapies that make use of ACE inhibitors or Ang II receptor type-1 antagonists [34,35]. The inflammatory response in kidney disease both during the acute and progressive phase is characterized by an interstitial infiltrate of leukocytes, which includes MCs [36,37,38,39]. The latter are localized predominantly in the interstitium, where they concentrate in areas close to tubules and blood vessels, but they never infiltrate glomeruli [39,40].

While little is known about the individual role of MC mediators such as histamine, tryptase and CPA, some significant progress has been made in the understanding of the role of chymase. In this review, we will discuss the role and actions of chymase in KD (Figure 1). While initially believed to contribute to pathogenesis, the accumulated data favor a more subtle view, indicating that chymase may also have beneficial functions. Hence, from a therapeutic standpoint, the pathophysiological context is important and may impose different strategies aimed either at inhibiting or strengthening chymase activity, in order to achieve therapeutic benefits in humans.

## 2. Mast Cell Infiltration and Kidney Disease

KD is characterized by the presence in human kidneys of numerous MCs (Figure 1). Their number can increase by up to 60-fold as compared to normal kidneys, where they are relatively sparse (0.5 to 1/mm^2^) [39,40]. In mice, MCs in normal kidneys are also relatively sparse, and the available evidence (with a few exceptions) shows that they do not significantly increase in short-term experimental models [41]. Note, however, that in mice and in humans MCs are present in significant numbers in the connective tissue of the kidney capsule surrounding the renal parenchyma both in normal and inflamed kidneys. The analysis of their degranulation phenotype indicated that there they are activated in renal disease models [31,42]. In some experimental models, MCs have also been shown to infiltrate draining lymph nodes (LN), where they play immunoregulatory functions (Figure 1) [43,44]. In humans, staining with tryptase and chymase-specific antibodies showed that kidney-infiltrating MCs were heterogenous, corresponding to the connective tissue-type MCs expressing both tryptase and chymase (MC_TC_) and the mucosal MC type expressing tryptase only (M_T_), although the ratio of the various MC subtypes varied with the renal disease [39]. In rejected kidney transplants, a third MC type, stained with anti-chymase only (M_C_), has also been found [45]. Although in most human nephropathies the increase in the number of MCs correlated with the concentrations of serum creatinine and with fibrosis, and thus with a poor outcome, functional studies of MCs in experimental models have yielded a more complex picture. Both disease-aggravating and disease-protective functions have been described, which was based not only on their kidney-infiltrating properties, but also on systemic actions and their capacity to perform immunoregulatory functions in kidney draining lymph nodes [39,40,41,44]. In addition, besides getting activated in the kidney, they are also present in the kidney’s surrounding connective tissues or kidney capsules, where they were shown to be activated [31,41,42]. Upon stimulation, they are able to release many inflammatory products either from sources prestored in secretory granules by degranulation or neosynthesized compounds such as prostaglandins and leukotrienes and a whole set of chemokines, cytokines and growth factors [46]. These are important in initiating tissue inflammatory and immunoregulatory responses, as well as tissue repair and remodeling responses that are relevant, including in KD [47,48].

## 3. Chymase as a Tissue-Ang II-Generating System in Kidney Disease

The RAS plays an important role in the maintenance of kidney homeostasis, but its dysfunction is also an important factor in the development of KD. Ang II is its main effector molecule. It causes vasoconstriction, increasing blood pressure, and regulates electrolyte homeostasis notably by promoting aldosterone release from adrenal glands [15] (Figure 1). Ang II also has potent proliferative inflammatory and profibrotic activities through its action through Ang I (AT1) receptors, leading to disease progression and renal fibrosis development [49]. By contrast, the interaction with less prominent, but inflammation-inducible AT2 receptors may have anti-inflammatory and anti-fibrotic functions [50]. Besides being generated through the RAS, chymase was identified as a major Ang II-forming enzyme in the human heart [12]. Further biochemical studies indicated that chymases from several species (humans, monkeys, dogs, hamsters, mice) can act as a tissue-generating system of ACE inhibitor-resistant Ang II [13,51]. Although initially attributed to α-chymases, these studies showed that structurally-related mouse β-chymases, and notably murine mMCP-4, was able to generate Ang II [13]. Both α-and β-chymases are thought to be the result of a duplication of a common ancestor during mammalian evolution [52]. The situation is more complicated in rats, as rat MC chymase rMCP-1 showed Ang II-degrading activity [53]. However, other enzymes not produced by MCs, such as rat vascular chymase (RVC) or rat mesenteric arterial bed elastase-2, may provide a relay in this species [54,55,56]. The possibility to have ACE-independent Ang II generation launched a series of studies evoking a possible role of chymase-dependent Ang II formation in various human tissues, such as the vasculature [51], heart [12] and kidneys after a high salt intake [57] and in patients with diabetic nephropathy [58]. The analysis of Ang II in human kidney lysates using mass spectrometry in the presence of an ACE inhibitor further showed that in normal kidneys more than 80% of Ang II generation was ACE-dependent, while this proportion was reversed in kidneys from CKD patients, supporting a predominant role in pathology [59] (Figure 1). The inhibitors of both ACE and chymase blunted almost all Ang II generation [59]. While these data are completely in line with significant renal infiltration by chymase-positive MCs, such as observed in rejected kidneys [45], IgA nephropathy [60,61], diabetic [58,62] and hypertensive nephropathy [63], as well as in autosomal polycystic kidney disease [64], they may simply reflect the relative amount of ACE and chymase in tissues, without being functionally relevant. To circumvent this issue, a study investigated ACE-specific and chymase-specific Ang II generation by directly infusing ACE- and chymase-resistant peptides in a model of diabetic nephropathy (DN) using genetically obese leptin receptor-deficient (Lepr^db^ or db/db) mice exhibiting features of human DN [65]. By determining the vasoconstriction response of renal afferent arterioles, the authors clearly demonstrated a switch from an ACE-dependent generation to a chymase-dependent generation during the development of the disease [62]. In a parallel study, they additionally demonstrated the enhanced vascular chymase-dependent conversion of endothelin-1 from its proform in the diabetic kidney, another factor by which chymase may contribute to renal diabetic pathology [66] (Figure 1). In parallel with disease development, the expression of the mRNA of ACE declined, while chymase expression increased. Subsequent therapeutic studies confirmed that chymase inhibition was able to delay development of albuminuria in type-2 diabetes [67]. Another study tried to analyze Ang II generation in a model of auto-immune glomerulonephritis induced by the injection of antibodies against the glomerular basement membrane (GBM), by comparing mice deficient in mMCP-4 with WT mice [42]. The data showed that mMCP-4-deficient mice exhibited improved renal function, as shown by lower proteinuria, blood creatinine and blood urea nitrogen levels. A histologic analysis confirmed the less severe renal damage, revealing reduced deposits, glomerular and interstitial cellularity, and fibrosis scores in mMCP-4 knockouts. Supporting the proinflammatory role of mMCP-4, glomerular and interstitial macrophage and T cell infiltration as well as mRNA levels of proinflammatory cytokines (TNF and CCL2) were decreased in the mMCP-4 knockouts. Most importantly, the expression of the profibrotic peptide Ang II was also markedly downregulated in the mMCP-4-deficient kidneys [42]. Although no significant MC infiltration was observed in the time-scale of disease observation (up to 14 days), the authors noticed that kidney capsule MCs were entirely degranulated as mMCP-4 completely disappeared in the capsules of diseased WT mice, while the level of the FcεRI β chain, a membrane MC specific marker, did not change during disease development [42]. This suggests the possibility that the chymase released from the MCs present in the connective tissue surrounding the kidneys may actively participate in disease development and Ang II generation. The same authors tested the role of mMCP-4 in the UUO model of renal fibrosis induced by unilateral ureteral ligation. Due to the complete obstruction of urine flow, one can observe rapidly (within a few days) a well-identified series of cellular and molecular events that characterize the initiation and progression of renal fibrosis [68]. Surprisingly, in this model, mMCP-4 proved to be protective, as mMCP-4-deficient mice had increased fibrosis scores with higher levels of renal tubular damage, interstitial fibrosis and collagen deposition compared to the kidneys of WT mice [31]. These data are in line with similar data obtained using MC-deficient mice, also showing a protective role of MCs as a whole [69], albeit this was disputed in another study [70]. Further functional analyses of mMCP-4-deficient mice showed that this was due to an increased inflammatory response in the absence of chymase as interstitial fibronectin deposition was increased, and as a consequence also the inflammatory cell infiltrate, in particular pro-fibrotic T cells and macrophages. Indeed, mMCP-4 is known to efficiently degrade fibronectin [30]. While MC numbers in the kidney did not significantly increase, chymase-containing kidney-capsule MCs were degranulated, albeit the released chymase did not completely disappear as in the anti-GBM model. Concerning Ang II, no significant difference in local Ang II generation or systemic differences in blood pressure were noticed between WT mice and mMCP-4 knockouts. Hence, in this short-term fibrosis model, ACE-dependent Ang II may remain predominant. Results obtained by the same team concerning the functional role of chymase were different when they used the more long-term model of the partial obstruction of urine flow (pUUO), with a more slowly developing fibrosis [71,72,73]. In fact, in this model, even two months after surgery, fibrosis scores were still moderate, with the mice developing fibrosis more focally but showing markers of early fibrosis such as a smooth muscle actin [73]. In contrast to the complete UUO model, MCs aggravated the disease and fibrosis scores, and an intermediate phenotype was observed for mMCP-4-deficient mice, indicating that mMCP-4 chymase partially contributed to the phenotype. However, Ang II generation has not been examined, and it remains therefore unknown whether in this more long-term model the chymase-mediated generation of Ang II contributes to the pathology. Finally, chymase-generated Ang II may also deteriorate kidney function in cisplatine-induced acute renal failure. Besides being promoted by IL18 and aldosterone, the inhibition of chymase activity improved acute renal failure. As renal failure was also blocked by an AT2, but not an AT1, receptor antagonist, this suggests that the action of chymase was to increase aldosterone, possibly causing an electrolyte imbalance (Figure 1) via the activation of AT2 receptors [74].

Taken together, studies conducted for many years have clearly shown that MC-released chymase represents an additional system to the classical RAS for the generation of Ang II, that could be involved in promoting kidney disease. Although the examination of Ang II-generating activity in kidney lysates could simply be a correlate to the significant MC infiltration in most kidney diseases, new functional studies confirmed the implication of chymase in Ang II-mediated pathophysiology. This may also partly relate to the pathophysiological context, as exemplified by the differences in Ang II implication observed in the glomerulonephritis and short-term fibrosis models.

## 4. Chymase in the Inflammatory and Tissue-Remodeling Response in Kidney Disease

The inflammatory process initiated after renal injury is destined to restore homeostasis by inducing a complex series of responses that includes the activation of the coagulation system to initiate hemostasis, inflammatory cell infiltration and inflammatory mediator secretion to eliminate infectious or injury-causing events. The released inflammatory factors and growth factors will also engender appropriate tissue repair mechanisms by initiating regenerative processes of injured tissue cells or tissue stabilization by mesenchymal replacement [75]. However, maladaptive insufficient or excessive responses and/or multiple iterative cycles of the injurious events will promote a progressive loss of function and CKD and eventually endstage renal failure when >70% of kidney parenchyma are lossed. Although this process is complex, involving numerous inflammatory cells and products, the previous characterization of chymase function indicates that this protease constitutes an integrative component in this process.

Chymase may play opposite roles in the coagulation process, being able to degrade both fibrinolytic plasmin [29,34] and fibrin-generating thrombin [28,29,30] (Figure 1). Additional data draw an even more complex picture. Comparing WT and MC-deficient mice, it has been shown that MCs limit fibrin deposits, thereby being protective in a model of autoimmune glomerulonephritis [76]. This was attributed to their ability to maintain high levels of tissue plasminogen activator (tPA) known to be released from MCs [77], as well as a urinary-type plasminogen activator (uPA) known to be activated by MC tryptase [78]. By contrast, the analysis of MC chymase in this model revealed a disease-aggravating role, as mMCP-4-deficient mice exhibited an attenuated disease [42]. A mechanistic investigation revealed that fibrin deposits in MC-deficient mice were not different from those in WT mice after disease initiation, excluding the profibrinolytic and profibrinogenic activities of mMCP-4 in this context. Further investigations attributed the disease-aggravating function to the local generation of proinflammatory Ang II, as already described above [42]. Therefore, the involvement of chymase in the modulation of fibrin deposition may depend tightly on the context of MC activation.

Another important function of chymase may be its ability to enable tissue remodeling and fibrosis development either by directly activating proforms of enzymes such as MMP2 and MMP9, able to degrade extracellular matrix (ECM) proteins, or by directly degrading matrix proteins such as fibronectin and collagen IV, or by activating/generating profibrotic inflammatory products such as TGFβ and Ang II [11] (Figure 1). While the role of chymase in local Ang II generation has been discussed in detail above, evidence has been provided that mMCP-4 can directly degrade ECM proteins. Early studies in 1981 revealed the susceptibility of soluble and matrix fibronectins to degradation by MC chymase [79]. The fibronectin-degrading activity was confirmed in mMCP-4-deficient mice, where certain tissues showed a markedly increased staining for fibronectin at the steady-state [25,30]. In agreement with these results, under inflammatory conditions in asthma, smooth muscle cells adhered less efficiently to the fibronectin ECM matrix in WT *versus* mMCP-4-deficient mice, which could be a means to relieve the mechanical transmission of tension from smooth muscles to the surrounding ECM [80]. Fibronectin degradation fragments were also shown to be proapoptotic for smooth muscle cells (SMC) [81], and thus may contribute to limiting the SMC layer built-up in asthmatic disease observed in mMCP-4-deficient mice [82]. mMCP-4 also promoted a favorable outcome after traumatic spinal cord injury by preventing exacerbated scar formation, by degrading fibronectin and the type-IV collagen that can accumulate excessively in the scar [83]. Concerning renal disease, in the UUO fibrosis model, the analysis of WT and mMCP-4-deficient mice showed that WT mice had lower interstitial fibronectin deposits and hence less infiltration of pro-fibrotic T cells and macrophages, thereby limiting fibrosis [31]. As mentioned above, local Ang II generation was secondary in this model, as no differences were noted for this parameter between WT mice and mMCP-4 knockouts, probably because ACE-dependent Ang II remained predominant. By contrast, in the partial pUUO model, where fibrosis development occurs more slowly, mMCP-4 aggravated the disease, albeit in a less pronounced manner than MCs as a whole [73]. Yet, fibrosis development and the expression of αSMA induced by TGFβ, a marker of mesenchymal transition, were enhanced after the pUUO procedure in WT and partially in mMCP-4-deficient mice, suggesting that MC involvement implicates mMCP-4-dependent and independent mechanisms. Supernatants of IgE-activated MCs contained substantial TGFβ-like activity, promoting the expression of αSMA in renal tubular cells in vitro [73]. However, in this study, it was not directly investigated whether the partial action of mMCP-4 was related to the activation of the proform of TGFβ. This feature was previously evidenced in vitro [21,22] and in vivo in a model of bleomycin-induced lung fibrosis based on the use of chymase inhibitors [84]. Chymase has also been described as an important activator of matrix metallo-proteinases zymogens, notably by generating active MMP2 and MMP9 [24,25,85], but also by inactivating the natural tissue inhibitor of metalloproteinase (TIMP)-1 bound to MMP9 [86]. The implication of these enzymes in renal disease is complex, with reported beneficial and detrimental effects depending both on the type of disease and the timing of intervention [87]. Presently, however, no study has examined the role of the chymase-mediated activation of MMPs in KD. Thus, chymase may act in multiple ways on fibrosis development: through the direct degradation of ECM components, the activation of ECM-degrading enzymes or the activation of profibrotic mediators (Figure 1). The balance between these actions, which depends on the context of MC activation, may determine the end-result of chymase involvement.

Besides interfering in the tissue remodeling response after an inflammatory event, human chymase was shown to directly cleave a restricted set of cytokines, which include the alarmins IL18 and IL33 as well as IL15, an important cytokine in T and NK cell homeostasis [32]. While human chymase did not cleave TNF, murine mMCP-4 present in lysates of peritoneal MC from WT but not mMCP-4-deficient mice was shown to degrade transmembrane and (albeit to a somewhat lesser extent) soluble TNF (Figure 1). This protected mice from an excessive inflammation in an experimental model of sepsis [88]. mMCP-4 was reported to increase the levels of cytokines in the intestine in an experimental model of infection with the parasite *Giardia intestinalis,* supporting a regulatory function [89]. In the same line, in a model of acute kidney injury induced by cisplatin, MCs aggravated the disease-enhancing TNF production by the rapid release of TNF, which in consequence enhanced the injury-associated inflammatory response and leukocyte recruitment [90]. However, the implication of mMCP-4 was not studied in this model.

Except for the possible role in the activation of pro-TGFβ mentioned above, no study has so far reported any effect on the role of chymase in alarmin/chemokine/cytokine degradation in KD.

Another important function of chymase could be its ability to cleave cell adhesion and junction proteins [91]. Indeed, the mMCP-4 chymase-dependent disruption of the claudin 4-dependent tight junction proved to enhance injury in a thermal injury model in the skin [92]. Another epidermal desmosome target for mMCP-4 has been identified in a mouse model of bullous pemphigoid induced by autoimmune Abs against the epidermal hemidesmosome transmembrane protein BP180 that was cleaved by mMCP-4 [93]. Similar observations were also made in the human bronchial epithelium, where the application of chymase to intact epithelial cell layers reduced the expressions of several cell adhesion proteins (occludin, claudin-4, ZO-1, E-cadherin), contributing to a loss of bronchial epithelial cell layer integrity, one of the prominent features of bronchial asthma [94]. In the intestine, while under homeostatic conditions, the ability of chymase to cleave claudin-3 contributes to maintaining intestinal permeability [95], which could also lead to intestinal epithelial barrier dysfunction in a pathological context [96].

Concerning acute KD, two recent studies on renal ischemia reperfusion injury (IRI) revealed opposing effects of MC and mMCP-4 [10,97]. While MCs aggravated the disease in this experimental model of IRI, especially during the early acute phase (up to 48 h) after reperfusion, mMCP-4 exhibited a protective function. In this model, it became evident that blunting the renal blood flow followed by reperfusion resulted in a systemic MC activation measurable by increased mMCP-1 chymase levels in the blood and by distant inflammatory actions of mMCP-4 in the hind paws [10,97]. Using an inducible model of MC depletion [98] when depleting MCs before injury, the associated inflammatory response increased, leading notably to an enhanced pathologic neutrophil infiltration and inflammatory cytokine/chemokine production [97]. When MCs were depleted past the acute phase, i.e., after 48 h, their depletion no longer influenced the disease, indicating that their action is in the early phase of this model [97]. When mMCP-4-deficient mice were examined, it was noted that, contrary to the situation in MC-deficient mice, the inflammatory neutrophil infiltration was less prominent, leading to a more controlled inflammatory response [10]. Interestingly, this could be attributed to the ability of both human chymase and mMCP-4 to cleave a certain number of adhesion molecules involved in neutrophil extravasation, such as CD162 (PSGL-1), CD54 (ICAM-1), P and E-Selectin [10] (Figure 1). Furthermore, chymase also blocked inflammatory neutrophil activation responses by a mechanism which has yet to be elucidated. These studies clearly shows that the proinflammatory mediator secretion by MCs can be fine-tuned and counterbalanced by mediators with anti-inflammatory activities released at the same time. In the described renal IRI model, mMCP-4 acts as an internal brake to limit the consequences of MC activation, which, overall, is disease-aggravating.

## 5. Chymase Inhibitor Studies in Kidney Disease

Targeting chymase could have important therapeutic interest. Therefore, besides analyzing the chymase function through the use chymase-deficient mice, some in vivo studies have also employed chymase-specific inhibitors. Indeed, a whole series of selective chymase inhibitors have been developed for some decades, and their efficacy profile, selectivity over cathepsin G and action on non-human chymase have been recently summarized [11]. Since the initial description and use of chymase inhibitors to demonstrate the Ang II-forming activity in the human heart [99], many of the studies based on inhibitors have been performed in the context of heart disease [100]. Concerning KD, only few studies based on the use of chymase inhibitors have been published. Initially, these inhibitors were used to demonstrate the existence of an ACE-independent system of Ang II that involves chymase in kidneys. This was notably demonstrated in chronic renal ischemia hypertensive models in dogs [101] and rats [102], as well as in a high salt-induced hypertensive model in mice [20]. The oral administration of the chymase inhibitor TEI-F00806 in a model of streptozotocin-induced diabetes in hamsters (which induces diabetic nephropathy associated with renal chymase expression) was shown to ameliorate proteinuria and several other parameters of pathology, including the normalization of Ang II levels [103]. Similar results were obtained in the same model in rats using the chymase inhibitor (OPh)_2_ [104]. More recently, in a more long-term model, the administration of (OPh)_2_ over 8 weeks using minipumps in diabetic (db/db) mice retarded the onset of albuminuria and reduced mesangial matrix expansion in glomeruli. However, Ang II levels were not altered by such treatment [67]. Using the same mouse models, another study also showed that the chymase inhibitor JNJ-18054478 prevented the generation of the mature potent vasoconstrictor endothelin-1 from its precursor form, supporting the notion that intrarenal chymase-dependent ET-1 production contributes to the decline in function and progression to end-stage renal disease in patients with type-2 diabetes [66] (Figure 1).

## 6. Concluding Remarks

Research in the last two decades has made clear that MCs contribute to the development of KD, but may also in some instances exert protective functions. Upon activation, MCs release many different inflammatory compounds, among which chymase may represent a sizable amount. Interest in studying the role of chymase in KD has been motivated by its ability to generate Ang II, an important inflammatory component in KD development. While these studies have confirmed the implication of chymase in the latter, notably in diabetic nephropathy and possibly hypertensive KD, the studies in KO mice have also revealed that this may depend on the pathophysiological context. Indeed, chymase can play opposite roles on fibrin deposition, extracellular matrix degradation or cytokine production, depending on the context in which the MCs that release this protease have been activated. In addition, while chymase promotes Ang II-dependent inflammation in an experimental model of glomerulonephritis, this does not seem to be the case in the UUO model, an accelerated model of kidney fibrosis development, or in a model of acute kidney injury induced by ischemia reperfusion injury. Other parameters such as the ability of chymase to participate in tissue remodeling with activation of proenzymes and cytokines and the cleavage of adhesion receptors may also be taken into account, as well as the ability of MC-released chymase to act systemically after reaching the circulation, or by its action in draining lymph nodes. A summary of the various findings in this review is presented in Figure 1 and further summarized in Table 1. Interestingly, these studies have also revealed that chymase may counteract inflammatory mediators released by MCs, even when released from the same compartment (i.e., secretory granules), supporting the notion that an inflammation is regulated by both pro- and anti-inflammatory mechanisms that can be summoned simultaneously [105].

## Figures and Tables

**Table 1:** Summary of the role of chymase in kidney diseases.

KD or KD Model	Type of Analysis of Chymase Activity	Mechanistic Role of Chymase and Relevant Reference (s)
db/db model of diabetic nephropathy	Infusion of ACE and chymase-resistant peptides	switch from ACE-dependent Ang II generation to a chymase-dependent generation during disease development [65]
db/db model of diabetic nephropathy	vasoconstriction response of renal afferent arterioles	enhanced vascular chymase-dependent conversion of endothelin-1 [62]
db/db model of diabetic nephropathy	chymase inhibition	delay in albuminuria development [67]
db/db model of diabetic nephropathy	chymase inhibition in mice and rats	Delayed onset of albuminuria, reduced mesangial matrix expansion in glomeruli, no effect on Ang II levels
streptozotocin-induced diabetes in hamsters	chymase inhibition in mice and rats	Improves proteinuria and normalizes Ang II levels [103,104].
anti-GBM glomerulonephritis model	mMCP-4-deficient mice	proinflammatory role of mMCP-4, decrease of Ang II levels in anti-GBM kidneys
Complete UUO model of renal fibrosis	mMCP-4-deficient mice	anti-inflammatory role of mMCP-4, increase in fibronectin deposits and profibrotic cell infiltrate (T cells, macrophages), no effect on Ang II levels with ACE-dependent generation remaining predominant [30].
Partial UUO model of renal fibrosis	MC-deficient (Wsh/Wsh mice) mMCP-4-deficient mice	intermediate pro-fibrotic phenotype when compared to MC-deficient mice, possible participation in the generation of mature TGFb [73]
cisplatine induced acute renal failure	chymase inhibition, use of AT1 and AT2 receptor antagonists, measurement of aldosterone levels and mMCP-4-deficient mice	pro-inflammatory role of mMCP-4, increase aldosterone causing an electrolyte imbalance via activation of AT2 receptors, [74] and generation of TNF [90]
renal ischemia reperfusion injury	MC-deficient (RBM mice) and mMCP-4-deficient mice	MC-deficient mice show a proinflammatory phenotype while mMCP-4 acts anti-inflammatory by controlling neutrophil infiltration through cleavage of cell adhesion molecules (PSGL1, E/P Selectin, ICAM-1) involved in margination [9]

## Data Availability

Data sharing not applicable.

## Abbreviations

MC	mast cell
mMCP	murine mast cell protease
RVC	rat vascular chymase
ACE	Angiotensin-converting enzyme
Ang I	Angiotensin I
Ang II	Angiotensin II
AT1/2	angiotensin 1/2 receptor
CKD	chronic kidney disease
db/db	diabetic mice, genetically obese leptin receptor-deficient (Lepr^db^)
DN	diabetic nephropathy
ECM	extracellular matrix
ET1	endothelin-1
GBM	glomerular basement membrane
IRI	ischemia reperfusion injury
KD	kidney disease
pUUO	partial unilateral ureteral obstruction
RAS	renin-angiotensin system
TGF-β	transforming growth factor-β
TIMP	tissue inhibitor of metalloproteinase
UUO	unilateral ureteral obstruction
WT	wild-type
